# A Minimum Set of Physiological Parameters to Diagnose Obstructive Sleep Apnea Syndrome Using Non-Invasive Portable Monitors. A Systematic Review

**DOI:** 10.3390/life11111249

**Published:** 2021-11-17

**Authors:** Ángel Serrano Alarcón, Natividad Martínez Madrid, Ralf Seepold

**Affiliations:** 1 School of Informatics, Reutlingen University, Alteburgstr. 150, 72762 Reutlingen, Germany; natividad.martinez@reutlingen-university.de; 2 Institute of Digital Medicine, I.M. Sechenov First Moscow State Medical University, 2-4 Bolshaya Pirogovskaya st., 119435 Moscow, Russian Federation; ralf.seepold@htwg-konstanz.de; 3 HTWG Konstanz, Department of Computer Science, Alfred-Wachtel-Str. 8, 78462 Konstanz, Germany

**Keywords:** obstructive sleep apnea, OSA, sleep disorders, non-invasive monitors

## Abstract

**Introduction.** Despite its high accuracy, polysomnography (PSG) has several drawbacks for diagnosing obstructive sleep apnea (OSA). Consequently, multiple portable monitors (PMs) have been proposed. **Objective.** This systematic review aims to investigate the current literature to analyze the sets of physiological parameters captured by a PM to select the minimum number of such physiological signals while maintaining accurate results in OSA detection. **Methods.** Inclusion and exclusion criteria for the selection of publications were established prior to the search. The evaluation of the publications was made based on one central question and several specific questions. **Results.** The abilities to detect hypopneas, sleep time, or awakenings were some of the features studied to investigate the full functionality of the PMs to select the most relevant set of physiological signals. Based on the physiological parameters collected (one to six), the PMs were classified into sets according to the level of evidence. The advantages and the disadvantages of each possible set of signals were explained by answering the research questions proposed in the methods. **Conclusions.** The minimum number of physiological signals detected by PMs for the detection of OSA depends mainly on the purpose and context of the sleep study. The set of three physiological signals showed the best results in the detection of OSA.

## 1. Introduction

Nowadays, sleep disorders are divided into seven categories according to the prevailing international classification of sleep disorders (ICSD-3): sleep breathing disorders, central hypersomnia, insomnia, isolated symptoms, parasomnias, sleep movement disorders, and circadian rhythm disturbances [1]. Within this classification, obstructive sleep apnea (OSA) is one of the most common sleep disorders [2]. OSA is caused by successive events of partial reduction (hypopnea) or total reduction (apnea) of the upper airways; this fact can lead to several issues such as arousals, hypoxia, and fragmentation of sleep [1,2,3]. Despite being one of the most common sleep disorders, OSA screening techniques are unavailable for many patients. Some studies state that up to 75% of patients remain undiagnosed and untreated [4]. This is due to several facts, but the most relevant is that the gold standard for OSA diagnosis is polysomnography (PSG). PSG is known to be the best performing OSA detection technique in terms of accuracy. However, PSG has several drawbacks that contribute to the delay in the diagnosis of many patients. First, PSG requires the patient to stay overnight in a sleep lab setting, and second, the study must be supervised by a physician using multichannel monitoring. These two requirements imply that PSG is expensive and time-consuming since a trained technician is necessary, along with the availability of a sleep laboratory to proceed with the clinical evaluation of OSA [5,6,7].

Considering the pitfalls derived from PSG, several OSA screening approaches have been proposed as an alternative to PSG, such as questionnaires and home sleep apnea tests (HSAT). Questionnaires such as the Berlin questionnaire, STOP-BANG, or NoSAS have been developed to evaluate and screen patients with OSA [8]. Nonetheless, according to the American Academy of Sleep Medicine (AASM), it is recommended that questionnaires are not applied to detect OSA without the use of technical devices since sleep-apnea-focused questionnaires and clinical prediction rules lack sufficient diagnostic accuracy by themselves [9]. Therefore, the principal substitute for PSG seems to be the application of HSAT using a portable monitor (PM).

A large number of PMs to detect apnea have been developed, since the first classification of practice parameters concerning the use of PM devices to detect OSA was carried out by the AASM in 1994. Since this classification was released, the AASM has applied several updates to it; however, as a consequence of the exponential growth of technological advances in the clinical realm over the years, Collop et al. [10] proposed a new classification that covers all the features of PMs that do not fit the categorization elaborated by the AASM. This classification is based on sensors used to measure physiological parameters related to “Sleep”, “Cardiovascular”, “Oximetry”, “Position”, “Effort”, and “Respiratory” and is known as SCOPER classification.

In this classification, the AASM classified sleep studies into four types based on the number of channels or physiological signals on the PM: type I (standard PSG; attended), type II (minimum of seven channels; unattended), type III (minimum of four channels; unattended), and type IV (one or two channels; unattended) [5,7,11].

A large number of manufacturers have contributed to the development of commercial PMs for the detection of OSA over the years [12]. Currently, there are many different approaches to developing PMs for OSA detection based on both research and commercial projects [3,10,12]. It is clear that to classify a PM for the detection of OSA, it is necessary to consider the physiological signals collected by the medical device. Despite this fact and together with the AASM recommendation for HSAT, which indicates a minimum of three physiological signals (airflow, respiratory effort, and oximetry) in order to achieve adequate OSA detection, there is no consensus among researchers on the minimum set of physiological parameters that a PM for OSA detection should include. Therefore, this article aims to investigate HSATs to evaluate the physiological signals of PMs to find the best minimum combination of biomedical signals that achieve both excellent accuracy in diagnosis and as little intrusiveness as possible for the patient.

For the search for a minimum set of physiological parameters for OSA, a systematic review was performed. Before the review was conducted, both the main and the set of specific questions were formulated.

### 1.1. Review Questions

#### 1.1.1. Main Review Question (MRQ)

Is there a minimum set of non-invasive parameters measured to diagnose, detect or monitor sleep apnea by in-home PM?

#### 1.1.2. Specific Review Questions (SRQ)

1. Does the outcome (OSA detection) improve if the number of psychological parameters measured increases?
2. What are the main requirements for the application of an in-home medical device to diagnose sleep apnea?
3. Is there a set of minimum physiological signals that distinguish between detection of sleep and arousal?
4. How does it affect the outcome (OSA detection) when PMs do not include oximetry measurement?
5. What physiological signals are included in those PMs that meet the criteria of positive likelihood ratios (LR+) of ≥5 and sensitivities (Sen) of ≥0.825?

The criteria for the inclusion and exclusion of papers and searches in databases are explained primarily in the Methods section. In the Results section, all publications that meet the criteria are analyzed, focusing on the number of physiological signals used and the precision in the detection of OSA. Finally, the discussion and the conclusion include a general interpretation of the results and the most relevant information extracted from the analysis. It is important to note that, contrary to another systematic review of PMs for OSA detection, the study does not focus on the features of medical devices, but on the physiological parameters collected by them.

## 2. Materials and Methods

This review was carried out following the “The PRISMA 2020 statement”, an update of the preferred reporting items for systematic reviews and meta-analyses (PRISMA) guideline [13].

### 2.1. Eligibility Criteria

According to [10], a PM for OSA detection should have an LR+ of ≥5 and a sensitivity not less than 0.825 after in-lab PSG—an apnea-hypopnea index (AHI) of ≥5 [14]. However, as shown in several publications, the achievement of LR+ of ≥5 is complicated. As a consequence, the number of publications is reduced to the minimum to perform an analysis. Therefore, for this study, publications were only included, if they met the criteria of having a sensitivity equal to or greater than 0.825 at AHIs of ≥5.

Inclusion criteria are shown as following:

▪ *Study type*: randomized controlled and clinical trials, research and review articles, and conference publications, along with clinical guidelines.
▪ *Population:* studies with adult (>18) patients referred to sleep clinics with symptoms suggestive of OSA.
▪ *Grouping of studies:* Differentiation between review publications and any other type of publication at the end of the search.
▪ *Outcome:* set of physiological parameters of PM (preferably type III or type IV).
▪ Exclusion criteria are shown as following:
▪ The articles are not in English or German.
▪ Published data are not available.
▪ Studies are not related to monitoring or diagnosing OSA using a PM.
▪ Studies of which the publication dates are older than 10 years when the systematic review is performed (2011–2021). Collop et al. conducted a review of studies covering PMs published prior to 2011 [10].
▪ Studies where there are an underlying diseases and are not entirely focused on OSA. The accuracy of PMs for the detection of OSA may be affected, if there are comorbid medical conditions such as pulmonary disease, neuromuscular disease, or congestive heart failure.
▪ PM (preferably type III or type IV) with a sensitivity of <0.825.

### 2.2. Search Strategy and Information Sources

The searches were performed from April 2021 to June 2021. The PubMed query was as follows: (“home”[TITLE] OR “portable”[TITLE] OR “ambulatory”[TITLE] OR “device”[TITLE]) AND (obstructive sleep apnea OR hypopnea) AND (“detection” OR “diagnosis” OR “monitoring”). For ScienceDirect, the query used was as following: TITLE: (home OR portable OR device OR ambulatory) AND (“obstructive sleep apnea” OR hypopnea) AND (detection OR diagnosis OR monitoring). Finally, for the IEEE Xplore Digital Library search, the query used was as following: (“Full Text Only”: “obstructive sleep apnea” OR “Full Text Only”: hypopnea) AND (“Full Text Only”: detection OR “Full Text Only”: diagnosis OR “Full Text Only”: monitoring) AND (“Document Title”: home OR “Document Title”: portable OR “Document Title”: device OR “Document Title”: ambulatory).

Six hundred and sixty-six references (before removing duplications) were collected from the databases (241 from PubMed, 334 from ScienceDirect, and 91 from the IEEE). The records were downloaded in a text format, and after removing duplications, a total of 629 references were qualified for the data evaluation step. One hundred forty-four articles were selected after the evaluation of the publications by reading their titles and abstracts. Eventually, full-text reading took place, ensuring that the inclusion/exclusion criteria for the articles assessed were met. The number of the studies included in the systematic review was 58. Statistical methods were used to analyze 45 publications. The remaining 13 were used to answer the main/specific questions.

### 2.3. Selection Process and Data Extraction

The studies identified after the search explained in the above section were exported to the Mendeley free reference manager tool. Subsequently, duplicates were automatically identified and eliminated. The next step was to select the remaining publications, first by their titles and then by their abstracts. The titles and abstracts of non-duplicated publications were selected, and then all publications that did not relate to the topic were removed.

The final step was the full reading of all the publications. For this purpose, a predefined form was filled with the data from the publication (manually by a researcher). The fields in the form were as following: paper name, year, SCOPER classification, PM sensitivity, PM specificity, LR+, AHI, population, arousal detection, and type of device. Those publications that consisted of reviews of several PMs were classified independently and reserved for answering the specific questions stated in the Introduction section.

### 2.4. Assessment of the Risk of Bias

The PMs were classified according to the physiological parameters used for each independent study. This means that for publications using the same type of device, the variation proposed by Mendonça et al. [3] for the SCOPER classification was performed to avoid bias in the outcome. The primary purpose of this systematic review was to analyze the set physiological signals, not the features of the medical device itself.

### 2.5. Synthesis Methods

Statistical analysis was carried out using a suite of Python packages (Pandas, NumPy, Seaborn, Matplotlib, and SciPy). Statistical data visualizations were presented to aid in understanding the results. Statistics were performed using Sen and specificity (Spe) reported by PMs and their relation to the number of physiological parameters used. As far as possible, statistics were presented to explain the relevant information collected during the systematic review.

## 3. Results

### 3.1. Study Selection

**Figure 1:**
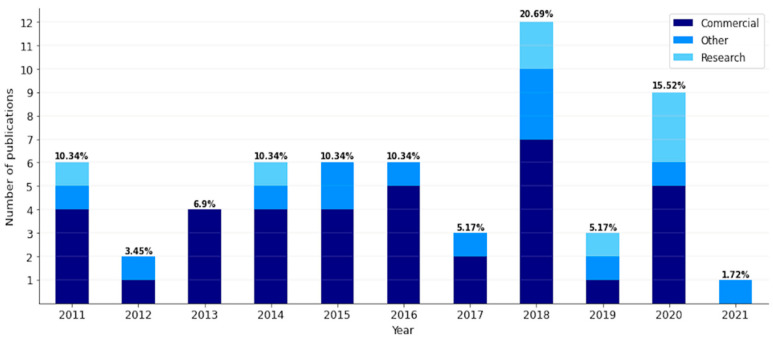
Bar-plots with the numbers of publications that met the inclusion/exclusion criteria. The terms “Commercial” and “Research” refer to the purpose of PMs. The term “Other” refers to the rest of the publications selected for this systematic review (e.g., book chapters and systematic reviews).

**Figure 2:**
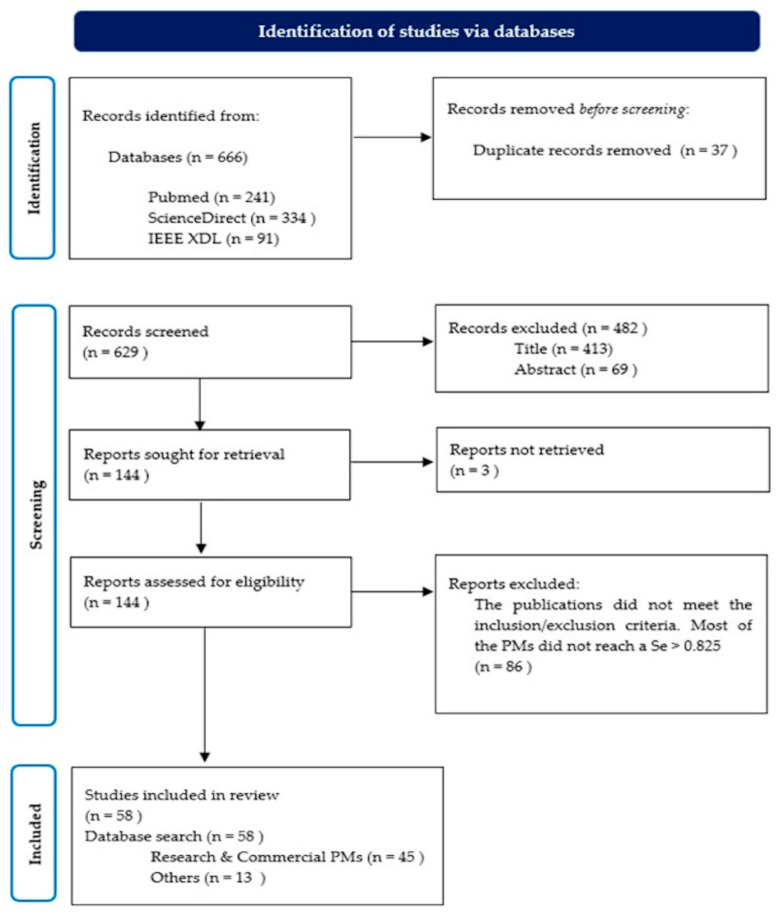
PRISMA 2020 flow chart for the selection of the entire set of the included publications.

Figure 1 shows the list of publications that met the inclusion/exclusion criteria and were selected for statistical analysis: the years of publications ranged from 2011 to 2021. The highest number of the selected articles was published in 2018 (20.69% of all publications), and the lowest number of the selected articles was in 2021 (1.72% of all publications). The research materials selected for this systematic review were divided into two main groups. A group was known as “Research and Commercial PMs” (see Figure 2) that included publications related to the use of standalone PMs. The publications of this group were used for statistical analysis. The other group, known as “Other(s)”, included publications that contained relevant information to answer the research questions stated in the Introduction and could not be included in the statistical analysis.

Figure 2 shows the whole process carried out throughout this systematic review. The numbers of publications included and excluded during each stage of the revision are also depicted.

### 3.2. Study Characteristics and Individual Publications

Table 1 includes all publications from the group “Research and Commercial PMs”. This table exposes information regarding PMs, such as metrics to evaluate their accuracy.

Table 2 (next page) includes all the publications from the group “Research and Commercial PMs”. In this table, a summary of each publication is also shown.

### 3.3. Synthesis Results and Questions of Interest

In this section, the central and specific questions stated in the Introduction section are answered and explained in detail.

#### 3.3.1. MRQ: Is There a Minimum Set of Non-Invasive Parameters Measured to Diagnose, Detect or Monitor Sleep Apnea by In-Home PM?

**Figure 3:**
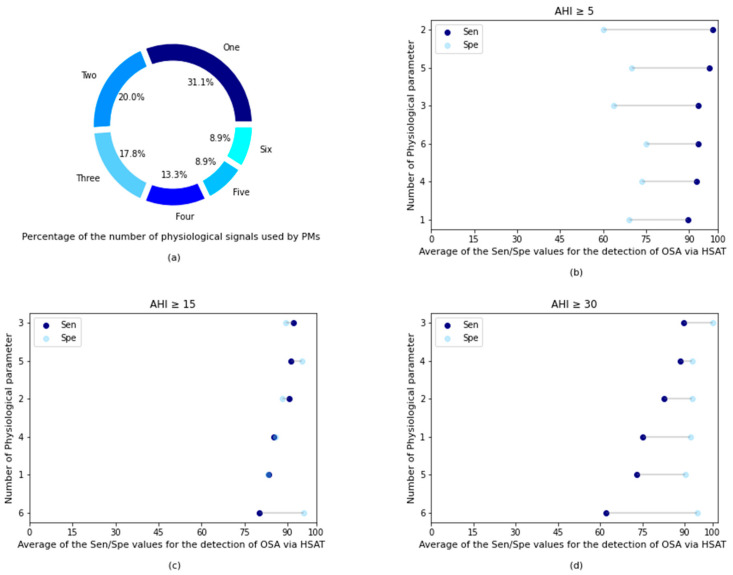
Statistical visualization of the results after data analysis. (**a**) Pie-chart with the percentages of physiological parameters used by each PM. (**b**) Average Sen/Spe for each PM based on the number of physiological signals for AHI ≥ 5. (**c**) Average Sen/Spe for each PM based on the number of physiological signals for AHI ≥ 15. (**d**) Average Sen/Spe for each PM based on the number of physiological signals for AHI ≥ 30.

The statistical analysis with the information extracted from the publications in Table 1 indicated that PMs can be implemented to collect up to six physiological parameters. Figure 3a shows that 31.1% of the PMs analyzed collected one signal only. The most relevant physiological parameter of these PMs was the respiratory signal; only three PMs did not collect respiratory signals, but cardiovascular or position signals. Single-channel PMs underperformed at all levels of OSA severity detection by other PMs measuring more than one signal. Only PMs with six or five channels worsened the results (in terms of Sen and Spe) for the detection of the severity of OSA compared to PMs with a single channel. In particular, for AHI of ≥15 and AHI of ≥30. On the other hand, 20% the PMs analyzed measured two physiological signals and obtained good results in detecting different levels of OSA severity. Together with the set of three signals (which represented 17.8% of PMs included in this study), the set of two physiological parameters reported the best results, always being one of the three best results for AHI of ≥5, AHI ≥15, and AHI of ≥30 (see Figure 3b–d).

The combination of four physiological signals (13.3% of the PMs included in this study) reported the second-best result for the detection of severe OSA, only worse than the set of three signs. It should be noted that all but one four-channel PM included oximetry. Finally, the sets of five and six psychological signals represented 8.9% of the total each. For both AHI of ≥5 and AHI of ≥15, the PMs that used five signals obtained the second-highest result in terms of Sen. Taking into account the results of the statistical analysis, the minimum set of signals that a PM should collect to obtain the best result when it comes to detecting OSA is two or three. In general, considering all levels of OSA severity, PMs with three channels showed the best results.

Regarding the group of publications known as “Other(s)”, five studies address this question. Hesselbacher et al. [11] state that the PM selection for OSA detection depends on many factors, along with the fact that on PMs with fewer signals, each independent channel becomes more important for the final clinical assessment. Berry et al. [60] conclude that PMs should measure airflow, respiratory effort, and blood oxygenation at a minimum. Dawson et al. [62] note that type III monitors performed better than type IV monitors in predicting AHI scores for OSA severity detection. Mendonça et al. [3] highlight the use of breathing analysis, alone or in combination with other sensors, as the method that yields the best results in detecting OSA. In addition to this, Mendonça indicates that a combination of oximetry and sound analysis can provide the best choice for respiratory analysis with minimal invasion. Jiang et al. [65] evaluated a set of physiological signals measured by PMs against PSG. A combination of four different sets of physiological signals was tested to obtain the AHI. The sets of parameters were as following: the first combination with a single nasal airflow, the second combination with nasal airflow + body activity, the third combination with nasal airflow + SpO2, and finally, the fourth combination with nasal airflow + SpO2 + body activity. Combination number four (S_3_O_1x_P_2_E_4_R_3_) drew the best agreement with PSG and obtained for AHI of ≥5, the highest Sen of 96.5%, and an Spe of 100%.

#### 3.3.2. SRQ-1: Does the Outcome (OSA Detection) Improve if the Number of Psychological Parameters Measured Increases?

As shown in Figure 3, the PMs included in this systematic review that collected more than three physiological signals to detect OSA did not report a significant improvement in their results for any of the AHIs. Only for AHI of ≥5 did the results of those PMs that collected five physiological parameters show better results than those that collected three. For AHI of ≥15 and AHI of ≥30, there was an improvement when looking at Spe for PMs that collected five and six physiological parameters, respectively, but no betterment was shown for Sen. In fact, PMs that measured at least six physiological parameters obtained the poorest results for AHI of ≥15 and AHI of ≥30. However, this may be due to several factors, such as the methodology when working with PMs that include more channels that are more similar to that proposed by the AASM, which can lead to more accurate results. Although it is more feasible than in this systematic review, the number of PMs that collected several physiological parameters equal to or greater than five represented the lowest number of PMs among the entire selection of PMs (17.8% of the total). Therefore, poor results in some PMs can affect Sen or Spe more significantly.

#### 3.3.3. SRQ-2: What Are the Main Requirements for the Application of an In-Home Medical Device to Diagnose Sleep Apnea?

A wide variety of publications in Other(s) highlight the importance of following the clinical guideline created by the AASM with a series of recommendations for the use of PMs for the detection of OSA at home [4,11,60,63,64,65,66,68]. In general, this guideline comprises three key factors: PM features, patients, and HSAT methodology. Regarding PMs, one of the most important is the minimal set of measured physiological signals (airflow, airflow, respiratory effort, and oxygen saturation). It is strongly recommended that the PM be easy to use. The PM setup is also crucial, as the potential for complications during HSAT increases with the complexity of the device. There should be an HSAT methodology available to assess the quality of the recordings, along with a sleep physician who supervises the sleep study. Lastly, with regard to the patient, there must be no comorbid conditions, since it could reduce the precision of the PM and present a high probability of moderate to severe OSA before the test [11,60,70]. Although such requirements were suggested by the AASM, as shown in this systematic review, most sleep studies on PM development agree on the fact that to devise a PM for OSA detection, there should exist a balance between the patient comfortability, the simplicity of the PM, and the precision of results.

#### 3.3.4. SRQ-3: Is There a Set of Minimum Physiological Signals That Distinguish between the Detection of Sleep and Arousal?

A total of seven publications from the group known as Other(s) address this question. According to Hesselbacher et al. [11], one of the most significant pitfalls with respect to the HSAT is that most PMs do not include electroencephalogram (EEG) or electrooculography (EOG) recordings. These physiological signals are needed to differentiate between the sleep-wake state and arousal. This fact has several implications. In principle, PMs cannot meet the arousal criteria recommended by the AASM to identify hypopnea, due to the impossibility to measure the total sleep time (TST) and the lack of the identification of respiratory events that meet the flow reduction criteria for hypopneas associated with microarousals instead of O_2_ desaturation [4,67]. As a consequence, the severity of OSA may be miscalculated as a lower detection of hypopnea due to the lack of EEG and the inclusion of respiratory events in the total recording time (TRT) rather than the TST, leading to a longer study time of sleep [4,11].

Although EEG or EOG is strongly recommended to detect awakenings, several alternatives have been proposed to work as surrogate signals in the detection of the sleep-wake state. Body movement, heart rates, and changes in sympathetic tone are surrogate signals used in the detection of sleep versus wakefulness [11,66]. Peripheral arterial tonometry (PAT) measures the volume of arterial pulse waves at the fingertip, and it can be combined with an accelerometer to determine wake/sleep. In this way, this combination detects an attenuation of the pulse amplitude that, combined with the acceleration of the pulse rate or an increase in wrist activity, indicates arousal or a respiratory event [11]. Other studies, such as Lachapelle et al. [67], state that pulse-oximetry-derived heart rate increases can be easily used as a surrogate marker of arousal in most type III PM. Vat et al. [4] conclude that drops in pulse wave amplitude (PWA) can be considered as a sensitive surrogate marker for EEG awakenings and used by PMs as a secondary criterion in the definition of hypopnea. Berry et al. [60] note that the combination of actigraphy and PAT signal has been used to determine estimates of wakefulness, non-rapid eye movement (NREM) sleep, and REM sleep, because the sympathetic tone characteristics of these sleep stages differ. On the other hand, Bianchi et al. [64] state that actigraphy works as a surrogate for EEG, because it overestimates sleep when movement is the only input due to quiet wake appearing similar to sleep. In [65], an accelerometer was used to calculate sleep time by subtracting the waking time from the time in bed. Light [66] suggests that using a single EEG lead may be possible to provide a helpful way to obtain the actual TST in an ambulatory HSAT.

From our statistical analysis with the publications of the group “Research and Commercial PM”, nine publications state that they detect arousals. The physiological parameters incorporated into these PMs ranged from three to six with a distribution of 33.33% for three signals, 33.33% for four signals, 22.22% for six signals, and 11.11% for five signals. The entire set of PMs was commercial devices, and seven of them included oximetry as one of their set of the collected physiological parameters. The two PMs which met the requirements of Sen of ≥0.825 and LR+ of ≥5 at AHI of ≥5 had a categorization of S3C2O1x (Sen = 95.8%, Spe = 100%, and LR+ = inf) [21] and P2R5A1 (Sen = 85%, Spe = 90%, and LR+ = 8.5) [30]. The lowest results were obtained with the PM that collected six physiological signals with a categorization of S_3_C_4_O_1_P_2_E_4_R_2_A_2_ (Sen = 88.2%, Spe = 72.7%, and LR+ = 3.23).

#### 3.3.5. SRQ-4: How Does It Affect the Outcome (OSA Detection) When PMs Do Not Include Oximetry Measurements?

The main inconvenience is related to the scoring of hypopnea and the classification of the severity of OSA. Pulse oximetry is required to calculate oxygen saturation levels [62]. Therefore, many research studies do not consider valid PMs that do not include the measurements of saturation signals for more than half the duration of the sleep study [2,3]. However, in this systematic research, both types of PM (concerning oximetry incorporation) were accepted, as PMs, which do not include oximetry, reported good results in Sen and Spe in the past [30,33,44].

**Figure 4:**
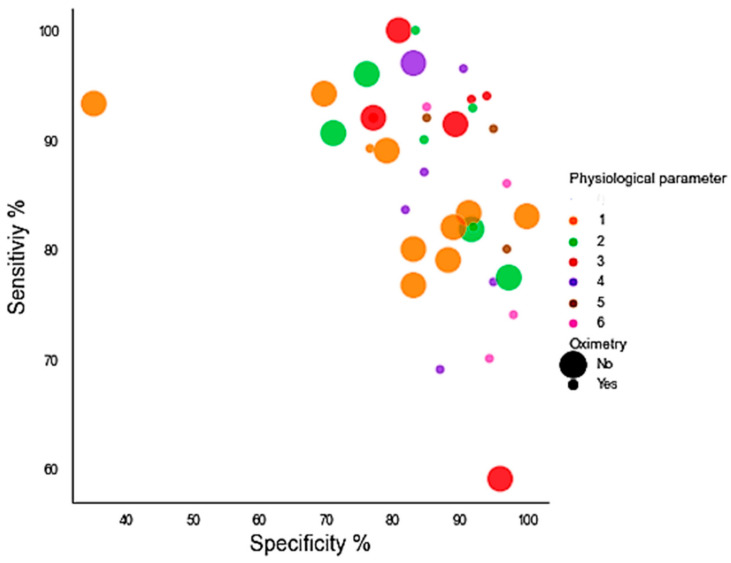
Scatter plots that show a relationship between the number of physiological parameters used by PMs and the inclusion of oximetry in terms of Sen/Spe.

Approximately 51% of the PMs from the publications included in Table 1 used oximetry. The rest of the PMs from the publications (49%) did not include oximetry as physiological parameters. Both sets of PMs (based on oximetry inclusion) reported similar results to those reported by Sen and Spe. The Sen and Spe results for these PMs, which included oximetry, were 86.65% and 89.06%, respectively. PMs which did not include oximetry obtained an Sen of 86.40% and an Spe of 82.24%. When looking at the results on the number of physiological parameters and the inclusion of oximetry, the results varied significantly, as shown in Figure 4.

On the basis of the results obtained from our statistical analysis, the inclusion of oximetry did not greatly influence the final result among all the PMs analyzed in this study.

#### 3.3.6. SRQ-5: What Physiological Signals Were Included in Those PMs That Met the Criteria of LR+ of ≥5 and Sensitivities (Sen) of ≥0.825?

Only four studies obtained results above the cut-off points set by Collop et al. [10] regarding the assessment of PMs to detect OSA. The only measure that did not include any of the PMs was effort, as shown in Table 3.

## 4. Discussion

The difficulty in diagnosing patients with OSA is a widely known problem. This problem lies mainly in the bottleneck caused by the waiting list of patients with a high probability of having OSA due to the time required to perform PSG. In addition to this, sleep studies are also expensive. As a solution, for some time now, more and more PMs have been developed to facilitate OSA detection.

The use of PMs for the diagnosis of OSA has been extensively studied and validated [5,6,12,14]. Therefore, in this systematic review, this issue has not been addressed. However, this study focuses on searching for the minimum set of parameters that a PM should collect for the accurate detection of OSA.

Although the AASM provides recommendations on PM use, they are not always followed. Based on the results obtained in this systematic review, the main objective when developing new PMs is usually to achieve the maximum patient comfort, together with adequate accuracy as close as possible to that obtained by PSG.

As a general rule, the AASM recommends for HSAT monitoring a minimum of three physiological parameters: airflow, respiratory effort, and SaO2 [9,62,70]. In theory, for monitoring such physiological parameters, the following sensors should be applied: generally, two sensors should be used for airflow measurement, an oronasal thermal sensor for apnea detection, and a pressure sensor for hypopnea detection. Regarding respiratory effort, respiratory inductance plethysmography (RIP) should be used. For SaO2 detection, oximetry is recommended [60,68].

This systematic review showed that not all PMs followed this recommendation. In contrast, the number of channels and the set of physiological signals collected varied widely. The statistical analysis of this study in Section 3.3.1 showed that a set of two or three channels can achieve the best results for OSA detection. Specifically, the set formed by three physiological signals was the one that yielded the best results. In this case, there was a correlation with what was declared by the AASM about the minimum set of physiological signals for accurate OSA detection. However, there was no concordance with the AASM recommendation on the nature of physiological signals that should constitute the set. This fact can be seen in Table 1, where PMs that recorded a set of three signals used different sets of channels from each other, obtaining similar results in OSA detection in terms of Sen and Spe. An example is that only two PMs of three channels coincided in the selection of the same set of physiological signals.

In addition to the aforementioned studies that validate the use of PMs for OSA detection, there are other studies on features that PMs should include, along with the set of signals they should collect. For example, Mendonça et al. [3] conclude that breath analysis, individually or with other combination of sensors, provides the best results in detecting OSA. Collop et al. [10] perform a comprehensive evaluation of the detection of PMs to detect OSA. It also provides a set of criteria for evaluating PMs that yield an accurate detection of OSA. Despite the number of publications, there is still no consensus on the minimum set of physiological signals that should be used to detect OSA using PMs. This systematic review aims to help to achieve this goal. However, this study has the limitation that it cannot provide a final set of physiological signals, as combinations are varied, as shown in the statistical analysis. This issue will be addressed in the future with the search for the best combination of signals for a three-channel PM, together with experimentation under the same study conditions. This is because in this type of research, the conditions of the experiments vary a lot between the studies using different PM configurations.

In general, in this study, a wide variety of PMs measured physiological signals, and the results obtained by these PMs in the detection of OSA have been analyzed. Once the signal measurement has been carried out, the methods used to assess whether the patient has OSA have not been evaluated in this analysis. Therefore, the calculation methods, algorithms, etc. have not been evaluated. It is also an interesting field to study in the future.

Another important aspect addressed in this study is the use of oximetry by PMs and the search for a minimum set of signals to detect arousals. Regarding the use of oximetry, there is a large division, with practically half of the PMs analyzed including oximetry and the rest which did not include it (see Section 3.3.5). There were no significant differences in the results, depending on whether oximetry was included or not. Regarding the minimum set of parameters for the detection of arousals, the sets that obtained the best results had a categorization of S_3_C_2_O_1x_ (Sen = 95.8%, Spe = 100%, and LR+ = inf) [21] and P_2_R_5_A_1_ (Sen = 85%, Spe = 90%, and LR+ = 8.5) [30]. They also met the criteria proposed in [10], which are an Sen of ≥0.825 and an LR+ of ≥5 at AHI of ≥5.

The fact that there is no agreement on the selection of metrics for the presentation of results in the scientific publications selected in this systematic review implies that many metrics obtained by PMs have not been taken into account. Sensitivity and specificity were chosen as study metrics to assess the diagnostic accuracy of PMs, because most publications include them in their analyzes, and Collop et al. [10] stated an Sen of >0.825 is one of the requirements for an adequate PM development. On the one hand, the cut-off points for AHIs (AHI of >5; AHI of >15; AHI of >30) were chosen, because most publications include them. On the other hand, these cut-off points are relevant indicators for distinguishing between mild, moderate, and severe OSA levels.

The SCOPER classification was selected, because it allows the inclusion of many PMs that remain unidentified with the proposal on the use of PMs for OSA detection by the AASM. Specifically, the specification made by Mendonça et al. [3] has been used in this analysis with relevant results. This variant of the SCOPER classification allows PMs to implement microphones for the collection of acoustic signals.

In this systematic review, a distinction was made between the two types of publications. First, those articles contained information on the validity of PMs in the detection of OSA. Second, those publications that included reviews, book chapters, or publications could help answer specific questions, but were not appropriate for inclusion in the statistical analysis, as they did not evaluate a single PM in particular. This was to avoid the removal of relevant information, since the publications contained in Table 2 could provide answers to specific questions.

In general, in response to the central question of this systematic review, it can be stated that the results are not significantly different from the guidelines developed by AASM in the number of physiological signals that are necessary to detect OSA. In contrast, the results of this review showed that there are a large number of signal combinations.

## 5. Conclusions

There is a large body of literature on the use of PMs to detect sleep disorders such as OSA. However, there is a less variety in terms of the physiological signals that should be used to develop such PMs. In this article, we carried out a systematic review of publications that include PM to detect OSA. We analyzed the number and characteristics of the physiological parameters used by the PMs mentioned above. Some aspects of arousal detection or alternative oximetry were also studied.

In summary, the answer to the main question of this systematic review after our statistical analysis is in agreement with the AASM recommendations. The set of three physiological signals for OSA detection is the best performing set. The set of the selected physiological parameters is affected by the type of sleep study, and the context in which it is found is greatly affected by the circumstances of the patient when using the device. On the other hand, increasing the number of physiological signals collected by PMs does not improve the overall results in OSA detection.

## Figures and Tables

**Table 1:** Publications (Research and Commercial PMs) that met the inclusion/exclusion criteria.

Publication	SCOPER Cat	Sen	Spe	AHI	Pop	Type Device
Jané, R. et al., 2011 [15]	R_5_A_1_	83	100	15	35	Research
Driver et al., 2011 [16]	S_3_C_4_O_1x_P_2_E_4_R_2_	97	67	5	73	Commercial
		80	97	15		
		70	100	30		
Nigro et al., 2011 [17]	R_2_	89.3	60	5	96	Commercial
		76.7	83	15		
		88.5	95.3	30		
Cheliout-Heraut et al., 2011 [18]	S_3_O_1x_P_2_E_2_R_x_	83.6	81.8	5	90	Commercial
				15		
				30		
Oktay et al., 2011 [19]	R_2_	90	76.9	5	53	Commercial
		79	88.2	15		
		66.7	95.9	30		
Ferré et al., 2012 [20]	S_2_C_4_O_x_P_2_E_x_R_2_	91	77	5	68	Commercial
		86	97	15		
		61	96	30		
Weimin et al., 2013 [21]	S_3_C_2_O1_x_	95.8	100	5	28	Commercial
		93.7	91.7	15		
		85.7	100	30		
Masa et al., 2013 [22]	R_2_	94	35	5	595	Commercial
		80	83	15		
Pereira et al., 2013 [23]	O_1x_P_x_E_1_R_2_	87	67	5	128	Commercial
		77	95	15		
		50	93	30		
Kobayashi et al., 2013 [24]	O_1x_C_4_P_2_R_5_	100	66.7	5	60	Commercial
		96.9	90.5	15		
Meissner et al., 2014 [25]	O_1x_R_2_E_1_	87.5	80	5	23	Commercial
Cairns et al., 2014 [26]	S_3_O_1x_P_2_E_1_R_2_A_x_	100	70	5	32	Commercial
		92	85	15		
Fredheim et al., 2014 [27]	C_4_O_1x_R_2_	93	71	5	99	Commercial
		94	94	15		
		90	1.0	30		
Garg et al., 2014 [28]	S_3_C_2_O_1_x	96	43	5	75	Commercial
		92	77	15		
Rodriguez-Villegas et al., 2014 [29]	R_5_A_x_	89	100	-	30	Research
Levendowski et al., 2015 [30]	P_2_R_5_A_1_	85	90	5	24	Commercial
		100	80.8	15		
de Vries et al., 2015 [31]	C_4_O_1x_R_2_	98.2	60.0	5	90	Commercial
		92.9	91.9	15		
Zou et al., 2015 [32]	C_4_O_1x_R_2_	80.28	95.45	5	93	Commercial
		87.04	84.62	15		
		94.87	92.59	30		
Alshaer et al., 2015 [33]	R_5_A_1_	98.1	82.8	5	135	Commercial
		77.4	97.3	15		
		65.6	100	30		
Gutiérrez-Tobal et al., 2016 [34]	O_1_	90.6	80	5	320	Commercial
		89.2	76.5	15		
		63.9	89.1	30		
Alakuijala et al., 2016 [35]	R_5_A_1_	93.3	35.1	15	211	Commercial
Nagubadi et al., 2016 [36]	S_3_O_1x_E_4_R_1_	69	87	15	71	Commercial
		87	66	30		
Ryan, C.M. et al., 2016 [37]	R_5_A_1_	90	84.6	15	23	Commercial
		100	85.7	30		
Álvarez et al., 2016 [38]	O_1_	94.2	69.6	15	320	Commercial
Durán-Cantolla et al., 2017 [39]	S_3_C_4_O_1_P_2_E_4_R_2_A_2_	88.2	72.7	5	28	Commercial
		70.0	94.4	15		
		100	92.6	30		
Xu et al., 2017 [40]	S_3_O_1x_P_2_E_1_R_2_A_x_	95	69	5	80	Commercial
		93	85	15		
		63	93	30		
Barbieri et al., 2018 [41]	R_5_	83.3	60	30	21	Commercial
Gumb et al., 2018 [42]	O_1_	85.9	76.5	5	178	Research
Mosquera-López et al., 2018 [43]	P_2_R_5_	81.82	91.7	15	14	Commercial
Massie et al., 2018 [44]	S_4_C_2_O_1x_P_2_	98	80	5	101	Commercial
		97	83	15		
		90	97	30		
Weinreich et al., 2018 [45]	P_2_R_5_	97.9	41.7	5	57	Commercial
		90.6	71.0	15		
Magnusdottir et al., 2018 [46]	C_3_	89	79	15	47	Commercial
Araújo et al., 2018 [47]	R_2_	81.8	61.5	5	35	Research
		83.3	91.3	15		
Bonnesen et al., 2018 [48]	P_2_A_1_	100	-	5	23	Commercial
		92.3	-	15		
Faßbender et al., 2018 [49]	O_1x_R_2_	100	44	5	48	Research
		92	77	15		
Mosquera-López et al., 2019 [50]	P_2_R_5_	88.9	76.5	15	14	Commercial
Chang et al., 2019 [51]	S_3_C_4_O_1x_P_2_E_1_R_2_A_x_	95	78	5	90	Commercial
		74	98	15		
		58	98	30		
Hayano et al., 2020 [52]	C_5_	82	89	15	41	Commercial
Fitzpatrick et al., 2020 [53]	P_2_R_5_A_1_	85	48	5	233	Commercial
		59	96	15		
Smith et al., 2020 [54]	O_1x_R_2_	82	92	15	100	Research
Mlynczak et al., 2020 [55]	S_3_P_2_A_1_	96	76	15	30	Research
Saha et al., 2020 [56]	S_3_P_2_R_5_A_1_	93.12	56.06	5	69	Commercial
		91.42	89.29	15		
		89.70	98.03	30		
Yamada et al., 2020 [57]	O_1x_P_2_R_1,5_A_1_	82.8	76	5	387	Commercial
		75.8	80.4	30		
Dzieciolowska-Baran et al., 2020 [58]	C_4_O_1x_E_x_R_x_	91	95	15	68	Research
Ferrer-Lluis et al., 2020 [59]	S_3_P_2_	90	-	15	13	Research

SCOPER cat: SCOPER categorization; Sen: sensitivity; Spe: specificity; AHI: Apnea-Hypopnea Index; Pop: population.

**Table 2:** Publications (Other(s)) that met the inclusion/exclusion criteria.

Publication	Objective	Type of Publication
Hesselbacher et al., 2011 [11]	Discussing the technical aspects and options available for portable home testing devices to diagnose sleep apnea.	Review article
Berry et al., 2012 [60]	Polysomnography (PSG), portable monitoring, and actigraphy when it comes to detecting OSA.	Book chapter
Shayeb et al., 2014 [61]	Systematic review and meta-analysis of comparative studies of level 3 versus level 1 sleep tests in adults with suspected sleep-disordered breathing.	Review article
Dawson et al., 2015 [62]	Comparison between the ability of the oxygen desaturation index (ODI) based on oximetry alone with a standalone pulse oximeter (SPO) and the respiratory disturbance index (RDI) to predict the AHI.	Research article
Vat et al., 2015 [4]	Investigating the performance of four different hypopnea scoring criteria, using 3% or 4% oxygen desaturation levels, with or without PWA drops as surrogates for electroencephalogram (EEG) arousals, and determine the impact of the measured versus the reported sleep time on OSA diagnosis.	Research article
Cooksey et al., 2016 [63]	Discussing society guidelines and recent research in the growing field of portable monitoring for OSA detection.	Review article
Bianchi et al., 2017 [64]	Studying the feasibility of home sleep apnea tests (HSAT) kits as they are known to underestimate the severity of sleep apnea, in part due to lack of sleep staging to provide total sleep time.	Research article
Jiang et al., 2018 [65]	Evaluating the combination modes of key physiological signals collected by portable sensor modules for OSA detection compared to PSG.	Research article
Light et al., 2018 [66]	Validating a single-channel frontal EEG for scoring sleep versus wake against full EEG during PSG and then examining the utility of adding this single-channel EEG to standard HSAT to prevent false-negative results.	Research article
Mendonça et al., 2018 [3]	Reviewing publications that show the performances of different devices for the ambulatory diagnosis of sleep apnea.	Review article
Lachapelle et al., 2018 [67]	Testing the hypothesis that scoring hypopneas using heart rate accelerations as a surrogate marker for cortical arousal (autonomic hypopnea; AnH) improves the accuracy of HSAT for OSA diagnosis, using PSG as the diagnostic gold standard.	Research article
Collop et al., 2020 [68]	HSAT overview.	Book chapter
Kwon et al., 2021 [69]	Summarizing recent results in the development of novel portable and wearable sensors for sleep monitoring.	Review article

**Table 3:** PMs that met the criteria of LR+ of ≥5 and sensitivities (Sen) of ≥0.825.

Publication	SCOPER Cat	AHI-5-Sen	AHI-5-Spe	Pop	LR+	LR−
Weimin et al., 2013 [21]	S_3_C_2_O_1x_	95.80	100.00	28	inf	99.04
Levendowski et al., 2015 [30]	P_2_R_5_A_1_	85.00	90.00	24	8.5	99.05
Zou et al., 2015 [32]	C_4_O_1x_R_2a_A_x_	80.28	95.45	93	17.6	99.15
Alshaer et al., 2015 [33]	R_5_A_1_	98.10	82.80	135	5.7	98.8

SCOPER cat: SCOPER categorization; AHI-5-Sen: sensitivity for AHI of ≥5; AHI-5-Spe: specificity for AHI ≥5; Pop: population; LR+: positive likelihood ratio: LR−: negative likelihood ratio.

## Data Availability

The authors will make available the data sets that support the conclusions of this article upon reasonable request.
